# Primary and Secondary Progressive Aphasia in Posterior Cortical Atrophy

**DOI:** 10.3390/life12050662

**Published:** 2022-04-29

**Authors:** Catherine Brodeur, Émilie Belley, Lisa-Marie Deschênes, Adriana Enriquez-Rosas, Michelyne Hubert, Anik Guimond, Josée Bilodeau, Jean-Paul Soucy, Joël Macoir

**Affiliations:** 1 Institut Universitaire de Gériatrie de Montréal, Montreal, QC H3W 1W5, Canada; catherine.brodeur.ccsmtl@ssss.gouv.qc.ca (C.B.); adriana.enriquez.ccsmtl@ssss.gouv.qc.ca (A.E.-R.); michelyne.hubert.ccsmtl@ssss.gouv.qc.ca (M.H.); anik.guimond.ccsmtl@ssss.gouv.qc.ca (A.G.); josee.bilodeau.ccsmtl@ssss.gouv.qc.ca (J.B.); 2 Université de Montréal, Montreal, QC H3T 1J4, Canada; jean-paul.soucy@mcgill.ca; 3 Centre de Recherche de l’IUGM, Montreal, QC H3W 1W6, Canada; 4 Département de Réadaptation, Faculté de Médecine, Université Laval, Quebec, QC G1V 0A6, Canada; emilie.belley.2@ulaval.ca (É.B.); lisa-marie.deschenes.cisssca@ssss.gouv.qc.ca (L.-M.D.); 5 McConnell Brain Imaging Centre, McGill University, Montreal, QC H3A 2B4, Canada; 6 Concordia University, Montreal, QC H4B 1R6, Canada; 7 Centre de Recherche CERVO (CERVO Brain Research Centre), Quebec, QC G1J 2G3, Canada

**Keywords:** posterior cortical atrophy, language impairment, logopenic variant of primary progressive aphasia, differential diagnosis, Alzheimer’s disease

## Abstract

Background: Posterior cortical atrophy (PCA) is a clinico-radiological syndrome characterized by a progressive decline in visuospatial/visuoperceptual processing. PCA is accompanied by the impairment of other cognitive functions, including language abilities. Methods: The present study focused on three patients presenting with language complaints and a clinical profile that was compatible with PCA. In addition to neurological and neuroimaging examinations, they were assessed with comprehensive batteries of neuropsychological and neurolinguistic tests. Results: The general medical profile of the three patients is consistent with PCA, although they presented with confounding factors, making diagnosis less clear. The cognitive profile of the three patients was marked by Balint and Gerstmann’s syndromes as well as impairments affecting executive functions, short-term and working memory, visuospatial and visuoperceptual abilities, and sensorimotor execution abilities. Their language ability was characterized by word-finding difficulties and impairments of sentence comprehension, sentence repetition, verbal fluency, narrative speech, reading, and writing. Conclusions: This study confirmed that PCA is marked by visuospatial and visuoperceptual deficits and reported evidence of primary and secondary language impairments in the three patients. The similarities of some of their language impairments with those found in the logopenic variant of primary progressive aphasia is discussed from neurolinguistic and neuroanatomical points of view.

## 1. Introduction

Posterior cortical atrophy (PCA), a clinical syndrome first described by Benson et al. [1], is characterized by predominant visuospatial and visuoperceptual deficits. PCA is accompanied by the impairment of other cognitive functions sustained by the bilateral occipito-temporo-parietal brain areas [2], including Balint syndrome (oculomotor apraxia, simultanagnosia, optic ataxia), alexia, and Gerstmann’s syndrome (agraphia, digital agnosia, acalculia, left and right confusion) [3]. In most cases, PCA is a visual phenotype of Alzheimer’s disease (AD) [4]. However, neuropathological studies have shown that, in some instances, other diseases, such as dementia with Lewy bodies [5], corticobasal degeneration [6], or prion disease [5], may be the cause.

In 2017, following a detailed review of the literature, an international multidisciplinary working group of researchers and clinicians proposed a consensus classification of PCA [7]. According to this classification framework, three distinct levels can be differentiated in PCA diagnosis. Classification level 1 established that PCA is a neurodegenerative disease not explained by other neurological or neuropsychiatric diseases and characterized by insidious onset and gradual progression. At this level, PCA is characterized by a progressive decline in visual processing and other posterior cognitive functions, while memory and language remain relatively unimpaired in the early stages. Neuroanatomically, classification level 1 is supported by evidence of predominant occipital, parietal, and occipito-temporal atrophy or dysfunction on brain imaging. Classification level 2 refers to PCA as PCA-pure or PCA with additional features (PCA-plus). To meet the criteria of PCA-pure, none of the core clinical criteria for corticobasal syndrome, the logopenic variant of primary progressive aphasia (lvPPA), or any other neurodegenerative syndrome can be present. PCA-plus refers to cases in which a core feature of any other neurodegenerative syndrome is present. Finally, to qualify for classification level 3, the underlying pathology of PCA, based on biomarker evidence, must show that PCA is attributable to AD, dementia with Lewy bodies, corticobasal degeneration, or prion disease.

### 1.1. Cognitive Disorders in PCA

The cognitive features associated with classification level 1 include the characteristics of Gerstmann’s syndrome as well as various visuoperceptual/visuospatial symptoms (space-perception deficit, simultanagnosia, object-perception deficit, environmental agnosia, optic ataxia, apperceptive prosopagnosia, homonymous visual-field defect) and sensorimotor symptoms (constructional dyspraxia, oculomotor apraxia, dressing apraxia, limb apraxia) [7]. To be considered classification level 1, at least three of these cognitive features must be present in the early stage of PCA and have a possible impact on the activities of daily living. In contrast, at this classification level, anterograde episodic memory, speech, language, executive functions, behavior, and personality must be unimpaired or at least relatively spared early in the disease [7]. However, as the disease progresses, these cognitive functions may also become significantly impaired.

In 2017, Maia da Silva et al. [8] described the cognitive disorders that are most frequently listed in PCA. Most of these disorders affect high-level visual abilities. Simultanagnosia, which refers to difficulty perceiving multiple objects when presented simultaneously, is believed to be present in over 90% of PCA cases [9]. It may appear in isolation before becoming part of Balint syndrome [5]. Maia da Silva et al. [8] also reported the presence of agraphia, acalculia, left–right disorientation, and digital agnosia in PCA. These impairments may present in isolation or as part of Gerstmann’s syndrome [10]. Ideomotor apraxia is also frequently reported in PCA [9], and visuospatial impairments may contribute to its functional origin [11]. Working memory is commonly affected in PCA [9], at a level of severity that can be greater than in AD [12] or lvPPA [13]. Verbal episodic memory might also be impaired in the early stages of PCA [14], presumably due to executive deficits [15]; however, this impairment is usually less severe in PCA than in AD. According to the consensus classification, executive functions must be relatively spared in classification level 1 [7]. However, a few studies have reported impairment of mental flexibility (e.g., [16]) in the early stages of PCA.

### 1.2. Language Disorders in PCA

The presence of language impairments was reported in the first descriptions of PCA; they were categorized as transcortical sensory aphasia characterized by anomia and deficits in language comprehension [1,17]. Moreover, some studies have highlighted frequent complaints regarding language abilities among patients with PCA. For example, Tang-Wai et al. [4] identified anomia as the initial complaint of over 80% of patients with PCA. Similarly, Migliaccio et al. [18] argued that about one third of patients with PCA had language complaints. However, PCA is largely known as a visuospatial and visuoperceptual syndrome, and language impairment has only been explored in a few studies.

In a study of 19 patients with PCA, McMonagle et al. [3] showed that agraphia and alexia were the most common linguistic symptoms. Meanwhile, the predominance of writing and reading difficulties in the clinical profile of PCA has been reported in other studies [9,11,16]. Agraphia has been described as one of the main clinical characteristics of PCA [1]. The manifestations of agraphia in PCA are linked to visuoperceptual/visuospatial impairment and are typical of spatial agraphia (substitution, omission and repetition of strokes, anomalous strokes, letter rotations, misplacement of letters on the page, inappropriate spacing between letters and words) [16,19]. A few studies have noted the presence of central agraphia, with patients presenting with surface agraphia [19], phonological agraphia [20], or impairment of the graphemic buffer [21]. With respect to reading, peripheral alexia is the most common form of reading impairment in PCA [22]. Patients may experience difficulties seeing [17] or identifying letters [23]. They may also present with alexia with simultanagnosia [24], neglect alexia [25], or letter-by-letter reading [26]. Central alexia characterized by difficulty reading nonwords [23,27] has also been reported.

When classifying patients according to aphasia types, typical profiles of anomic aphasia in 70% of the cases, Wernicke’s aphasia in 25%, and conduction aphasia in 8% have been identified [3]. In a retrospective study conducted with nine patients diagnosed with PCA, Magnin et al. [28] found anomia in spontaneous speech in 77% (7/9) of the patients. The difficulty in accessing lexical-semantic representations was not entirely due to visual impairments, since anomia was reported with similar proportions in a picture naming and a naming to verbal description and definition task. Magnin et al. [28] also reported impairment in sentence repetition (55%, 5/9) but not in word repetition. In a recent study, Tezloff et al. [29] explored the production of phonological errors in PCA and lvPPA using repetition, verbal fluency, and picture naming tasks. They showed that phonological errors are not only common in lvPPA but also frequently observed in PCA patients (55%, 15/27). 

A few studies have explored specific impairment of language processing in PCA. In a single case study of PCA, Steeb et al. [30] explored the impairment of semantic verbal fluency of nouns and verbs. Two other studies found specific impairment in the semantic processing of words related to quantity and space [31,32].

Meanwhile, in 2013, Crutch et al. [13] developed the most complete description of language functioning in PCA by comparing the performance of 15 participants with PCA, 7 participants with lvPPA, and 18 age-matched healthy participants using neurolinguistic assessment tests. Compared to the healthy participants, participants with PCA showed impairment in auditory input processing (prosody discrimination), repetition of nonwords, sentences, clichés, picture naming, letter and semantic verbal fluency, word spelling, and word and sentence comprehension. In contrast, no difference between the healthy and the PCA groups was noted on auditory–verbal minimal-pair-discrimination and word-repetition tests. With respect to spontaneous speech, significant differences in speech rate and word frequency were recorded between the two groups; however, the results were the same for the total number of words produced, the type–token ratio (number of different words/total number of words), and the number of word-finding pauses. Finally, participants with PCA performed worse than healthy participants but better than participants with lvPPA on every language test.

In summary, although PCA is essentially a visual syndrome, a few studies have reported language impairment, even in its early stages of progression. In most of these studies, the characterization of language was not the first objective. Many of them were limited to the description of deficits affecting written language in PCA [19,20,21,23,24,25,26,27]. The primary aim of other of these previous studies was to characterize the general cognitive profile of PCA [3,9,14] or to differentiate this profile from the cognitive profile of AD [16,22], so that language functioning was assessed superficially. Finally, very specific language abilities such as phonological processing [29], lexical access to nouns and verbs [30] and lexico-semantic processing of semantic categories [31] have been explored in a few other studies. Therefore, only very limited data are available on language impairment in PCA.

The functional origin of language impairment can be primary (i.e., language impairment due to deficits of linguistic processes) or secondary (i.e., impact of other cognitive impairment on language functioning). This functional origin of language impairment in PCA has not yet been described in the literature and deserves to be specifically explored.

Thus, the two main objectives of the present study were: (1) to provide an extensive description of the medical, cognitive and language profile of three new cases of PCA and, (2) to explore specifically the functional origin of their language impairment and, thus disentangle which impairment was caused by secondary (visuospatial and visuoperceptual processing, verbal short-term and verbal working memory, and executive functions) or primary (language) deficits. Their sociodemographic status and medical condition will be presented, followed by a description of their neuropsychological and neurolinguistic profiles.

## 2. Clinical Illustrations

Three individuals were recruited at the Institut Universitaire de Gériatrie de Montréal (IUGM). They were diagnosed with PCA by an experienced geriatrician (CB) using Crutch et al.’s [7] criteria.

### 2.1. Medical Clinical Profile

The participants’ demographic data and medical and general cognitive test results are presented in Table 1. The symptoms’ duration varied widely for the 3 patients (from eight and six years for P1 and P2, respectively, to only two years for P3). The duration of their symptoms was not correlated to the severity of impairment. This might be explained by different underlying neuropathologies (AD, Lewy-body disease, corticobasal degeneration, or even prion disease) and other contributors to the cognitive impairment, such as excessive alcohol intake.

#### 2.1.1. Patient 1

Patient 1 (P1) is a 63-year-old right-handed performance artist with eight years of education. He is of French origin and has lived in Quebec since 1998. He is a unilingual francophone who lives alone. According to his neurologist, his cognitive impairment, which has been present since around 2009, is relatively stable despite a slow decline in the Montreal Cognitive Assessment (MoCA) [33,34]. His main complaints, when assessed by the geriatrician in 2017, relate to visuoperceptual and visuospatial difficulties (e.g., difficulty putting on his coat, increased time required to prepare his scene performance) as well as difficulties finding words, reading, and writing. In 2016, an occupational-therapy assessment revealed the presence of dressing apraxia, spatial disorientation, dysexecutive impairment affecting divided attention, agraphia, and visuoperceptual impairment on the Motor-Free Visual Perception Test [35].

A medical examination performed in 2017 by the geriatrician revealed the presence of word-finding difficulties (especially for proper names), simultanagnosia, left and right confusion, digital agnosia, and acalculia. Structural neuroimaging (MRI) showed lesions compatible with chronic microvascular disease, while functional neuroimaging (FDG-PET) showed reduced metabolism in the right posterior occipital region and the bilateral posterior parietal cortex. Unfortunately, the functional imaging of P1 was done in another hospital and the data are no longer available.

#### 2.1.2. Patient 2

Patient 2 (P2) is a 74-year-old right-handed woman with 16 years of education. She is a unilingual francophone and a native Quebec French speaker. She retired from her position as a high school teacher 10 years ago. She lives alone in a residence for independent elderly persons. She was referred to the IUGM cognition clinic by a neurologist for a differential diagnosis between AD and lvPPA. For a year to a year and a half, the patient’s family noted cognitive changes affecting language abilities (word finding, comprehension of less frequent words, decrease in reading activities, difficulty writing), episodic memory, learning (using the television and voicemail), and instrumental activities of daily living and hobbies (cooking, managing household finances, playing Scrabble). P2′s main complaints relate to word-finding difficulties, loss of interest in reading, and difficulty with calculation.

**Figure 1:**
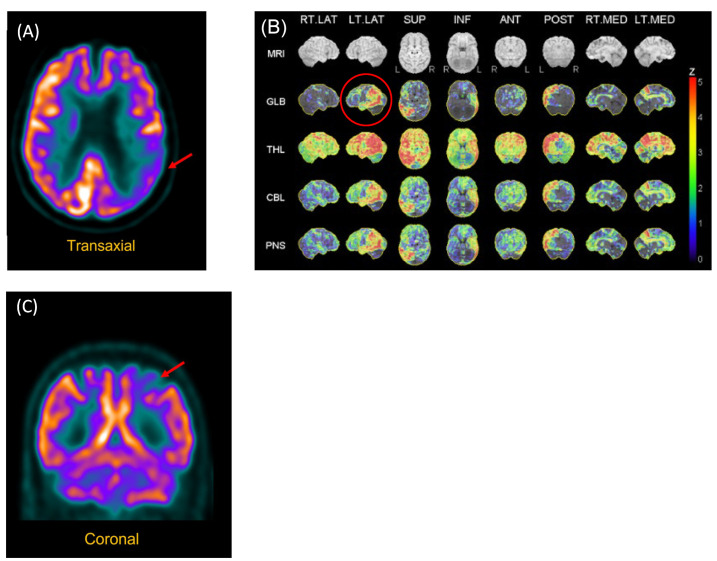
FDG-PET Scan of P2 and P3. (**A**) P2. Reconstructed slices showing hypometabolism of the left polymodal associative areas, especially the parieto-occipital regions, as well as asymmetry (left > right) of the primary visual areas. (**B**) P2. Statistical analysis of metabolism distribution as compared to marched normal database showing areas of hypometabolism in the left hemisphere, including in the left posterior temporal region (image circled in red). (**C**) P3. Hypometabolism of the left posterior parietal region.

The following problems were noted by the geriatrician during an examination performed in 2017: empty speech with word-finding difficulties manifested by vague terms and hesitations, calculation and number processing (comprehension of magnitude) difficulties, ideomotor apraxia, and digital agnosia. MRI showed white-matter periventricular hyperintensities and volume loss in the bilateral temporal lobes, while an FDG-PET scan showed hypometabolism of the left polymodal associative areas, especially the parieto-occipital regions, as well as asymmetry (left > right) of the primary visual areas (see Figure 1). 

#### 2.1.3. Patient 3

Patient 3 (P3) is a 64-year-old left-handed man (forced use of the right hand) with nine years of education. He works as a forklift operator and lives in a house with his wife. He is of Portuguese origin and has lived in Quebec since 1967. He is trilingual (Portuguese, French, and English). He was referred to the IUGM cognition clinic to investigate possible AD. The patient’s main complaint for about one year was increased difficulty expressing his thoughts and forgetting what he wants to say, without word-finding problems. His wife noted that his sentences were shorter and simpler, and that he often limited his responses to yes or no answers. Some work colleagues also expressed concerns about his language. The patient noted that his handwriting was less clear and that he had more difficulty forming letters and signing his name. At work, he had new difficulty parking forklifts. He did not think he had memory or orientation problems.

There were no focal signs on neurological examination apart from a slight decrease in spontaneous blinking, slightly slow horizontal and vertical saccades, and discreet hypertonia with cogwheeling at the left upper limb. There was no bradykinesia, and walking was completely normal without parkinsonism or dystonia. The cognitive examination performed by the geriatrician showed verbal aspontaneity and the production of short sentences but no word-finding problems on the Boston Naming Test [36]. Writing words and sentences under dictation was slow and possible only using capital letters. The patient was unable to sign his name in cursive letters. There were also signs of visuoconstructive apraxia, ideomotor apraxia, left and right confusion, digital agnosia, and acalculia for more complex mental operations (e.g., 12 × 5).

Structural neuroimaging (CT brain) was normal. However, functional neuroimaging (FDG-PET scan) showed hypometabolism of the left posterior parietal region (see Figure 1).

#### 2.1.4. Summary

The general clinical profile of the three patients was fairly consistent with level 2 (PCA-plus) of the classification framework proposed by Crutch et al. [7]. They presented many signs of PCA, along with features of other neurodegenerative syndromes, in particular with lvPPA. However, their clinical portrait was complicated, especially because of alcohol abuse (P2 and P3) and microvascular brain disease (P1 and P2). In all three patients, the cognitive decline, which mainly affected visual processing and posterior cognitive functions, was not explained by other neurological or neuropsychiatric diseases and was characterized by insidious onset and gradual progression. The patients all had complaints about language functioning, mostly related to difficulties finding words, reading, and writing. The patients were all referred for neuropsychology and speech-language pathology. Their detailed cognitive profiles will be described in the following sections.

### 2.2. Cognitive Profile

P1, P2, and P3 were administered a neuropsychological battery that included tests of executive functions (Trail Making Test A and B, Stroop Victoria, Tower of London test) [37,38,39,40,41], verbal short-term and working memory (Digit Span subtest from the Wechsler Adult Intelligence Scale) [42], episodic memory (12-item Buschke memory test, logical memory subtest from the Wechsler Adult Intelligence Scale) [42,43] visuospatial and visuoperceptual abilities (Visual Object and Space Perception Battery, Bells test) [44,45], sensorimotor execution (Batterie brève d’évaluation des praxies) [46], and objectification of Balint and Gerstmann’s syndromes. There were some differences in the tests selected by the two neuropsychologists who made the evaluation. The patients’ performance in all tests was compared to published normative data. The cognitive test results are presented in Table 2.

The degree of severity of the deficits was essentially based on clinical experience as well as on the following global criteria: 1.5 to 2 standard deviations (SD) below the mean standard score corresponds to mild impairment; 2 to 2.5 SD below the mean standard score corresponds to moderate impairment; >2.5 SD below the mean standard score corresponds to severe impairment.

As shown in Table 2, P1, P2, and P3 had very similar cognitive profiles with impairments affecting executive functions, short-term and working memory, visuospatial and visuoperceptual abilities, and sensorimotor execution abilities. Balint and Gerstmann’s syndromes were both found in the three patients. With respect to episodic memory, encoding and consolidation processes were unimpaired; meanwhile, the retrieval of encoded information was affected due to the executive impairment. This profile is largely congruent with studies in which cognitive functions were more extensively explored in individuals with PCA [8,9,11,14,15,16].

### 2.3. Language Profile 

P1, P2, and P3 underwent a neurolinguistic battery that included tests of semantic memory (picture-to-picture matching subtest of the BECLA battery, Pyramids and Palm Trees Test, word-to-picture matching subtest of the Montreal-Toulouse battery) [47,48,49,50,51], oral comprehension [48], picture naming [48,51,52], verbal fluency [53], repetition [48], narrative discourse [48], reading, and writing [48]. As for the cognitive assessment, there were some differences in the tests selected by the two speech-language pathologists who made the evaluation. The patients’ performance in the cognitive tests was compared to published normative data. The language test results are presented in Table 3.

The degree of severity of the deficits was based on clinical experience as well as on the same global criteria as for the cognitive assessment.

The linguistic portrait of the three patients supports the results of the few studies on language impairment in PCA. Comprehension abilities have not been extensively studied in PCA. However, in the present study, we found the preservation of comprehension at the word level (mild impairment in P2), while all three patients showed impairment related to sentences and complex instructions. Crutch et al. [13] reported impairment of not only sentence comprehension but also word comprehension (semantic processing of concrete and abstract words).

P1 and P2 showed anomia in picture naming, which has been reported in previous studies [2,13,28,29]. Consistent with Magnin et al.’s [28] findings, in the present study, word-finding difficulties were not exclusively due to visual impairments, since all three patients also presented with anomia in narrative discourse. The impaired performance in letter and semantic verbal fluency was hardly surprising considering the deficit of executive functions observed in the three patients, who were highly involved in these tasks. Putcha et al. [15] also suggested the executive origin of the impairment observed in verbal fluency in PCA.

As in the PCA patients studied by Magnin et al. [28] and Crutch et al. [13], repetition was impaired in P2 and P3 (not assessed in P1) for long sentences but not for words. Semantic processing was preserved in the two patients; they produced semantic substitutions, preserving the meaning of the sentences. Word omissions and phonological errors were also observed in their performances. This deficit must be linked to the impairment of phonological short-term memory in these two patients.

Crutch et al. [13], the only researchers to explore spontaneous speech in PCA, found significant differences between healthy participants and patients with PCA in terms of speech rate and word frequency. To the best of our knowledge, language abilities in narrative discourse have not been studied in PCA. However, in the present study, we showed that, in addition to word-finding difficulties, all three patients were impaired when involved in a narrative-descriptive scene description. This task is useful to assess various linguistic processes at the phonetic/phonological (e.g., production of phonological errors, hesitations, pauses), lexico-semantic (e.g., production of semantic errors, use of empty words), syntactic (e.g., mean length of sentences, syntactic errors, syntactic diversity) and pragmatic (e.g., cohesion, topic maintenance) levels [54]. In this task, they showed tangentiality (P1), reduction in speech rate and aspontaneity (P2 and P3), production of short but grammatical sentences (P3), and manifestations of word-finding difficulties (P1, P2, P3) (i.e., production of semantic, formal, and phonological errors).

As in most reports on language impairment in PCA, peripheral alexia was observed in all three patients in the present study. This impairment manifested in the production of visual errors (P1, P3), slowness (P2, P3), impaired word recognition (P3), line breaks, and word omissions (P3). Consequently, reading comprehension was affected in all three patients. This profile of peripheral alexia is largely congruent with the results of previous studies [3,9,11,16,17,23]. In addition, phonological alexia (difficulty reading nonwords) was noted in all three patients and identified in two previous studies [23,27].

Finally, the three patients in the present study showed peripheral as well as central agraphia. Peripheral agraphia is typical of PCA and usually manifests as spatial agraphia [16,19], mainly due to visuoperceptual/visuospatial impairment [55]. P1, P2, and P3 also exhibited spatial agraphia, which is characterized by the production of distorted letters and inappropriate spacing between letters and letters/words spatial misplacements on the page. A few studies [13,20,21] reported central agraphia, mainly characterized by the production of non-phonologically plausible errors (i.e., letter substitutions, omissions, additions, transpositions), and a negative effect of word length on performance. This writing pattern is suggestive of impairment of the graphemic output buffer [56], a functional localization proposed by O’Dowd and de Zubicaray [21] to explain the pattern of writing impairment in a patient with PCA. However, it should be noted that P3 also produced phonologically plausible errors, therefore indicating mixed agraphia.

## 3. Discussion

The clinical profile of all three patients was globally suggestive of the level 2 (PCA-plus) of the classification framework proposed by Crutch et al. [7]. Although the neuropsychological and neurolinguistic profiles were largely congruent with PCA, microvascular brain disease was found in P1 and P2. Alcohol abuse was another confounding factor in P2 and P3.

Many of the presented clinical signs—Balint syndrome, Gerstmann’s syndrome, and alexia—are typical and relatively specific of PCA. However, some features were also compatible with primary progressive aphasia (PPA), especially its logopenic variant (lvPPA). PPA is a heterogeneous neurodegenerative syndrome mainly characterized by a prominent difficulty with language, while other cognitive domains are not affected at the onset or early stages of the disease [57]. According to the 2011 recommendations for PPA diagnosis and classification [58], there are three main PPA variants: the nonfluent/agrammatic variant (nfvPPA), the semantic variant (svPPA), and the logopenic variant (lvPPA). While clinicopathological studies have shown that nfvPPA and svPPA are typically caused by frontotemporal degeneration pathology, lvPPA is most often caused by AD pathology [59], such as PCA. The following core features are essential to a diagnosis of lvPPA: (1) the presence of anomia in spontaneous speech and confrontation naming and (2) the impaired repetition of sentences and phrases. At least three of the following features must also be present: (1) the production of phonological errors, (2) the preservation of semantic memory, (3) the preservation of articulation and prosody, and/or (4) the absence of frank agrammatism.

P1, P2, and P3 had anomia in spontaneous speech (narrative discourse), which was marked in P2 and P3 by the production of phonological errors. Confrontation naming was impaired in P1 and P2. The fact that word-finding problems were noted in narrative speech rules out the exclusive visual origin of anomia. As we proposed for lvPPA [60], we suggest that disruption in the activation of the phonological forms of words is responsible for anomia and the production of phonological errors in PCA. Some studies have also shown that verbal short-term memory impairment contributes to spoken word-production impairment in lvPPA [61]; this cognitive deficit was present in P2 and P3 in the present study. With respect to repetition, individuals with lvPPA show significant long sentence impairment due to reduced verbal short-term-memory capacities [62]. In this task, their performance is marked by word omissions, semantic substitutions (replacement of one or more sentence words with words having similar or closely similar meanings), and phonological errors [63]. In the present study, a similar impairment of the repetition of long sentences was observed in P2 and P3 (not assessed in P1). The remaining criteria for lvPPA (production of phonological errors, preservation of semantic memory, preservation of articulation and prosody, and/or absence of agrammatism) were also fulfilled in all three patients in the present study.

Previous studies have reported an overlap in the clinical profiles of lvPPA and PCA. For example, Crutch et al. [13] compared the performance of patients with PCA and lvPPA and found impairment of a similar magnitude on tests of auditory input processing (auditory discrimination of words), repetition (nonwords and sentences), and digit span (forward and backward). According to the authors, this result suggests that language impairment in PCA is characterized by difficulty in the manipulation and retrieval of phonological information due to weakened verbal short-term memory, as is the case in lvPPA. Meanwhile, in eight of the nine patients with PCA they studied, Magnin et al. [28] found a logopenic syndrome, which was characterized by anomia in spontaneous speech and picture naming, reduced performance in letter and semantic fluency, and length-dependent deficits in sentence comprehension and sentence repetition. The patient without language impairment was the only one with an isolated right posterior cortical abnormality. Fitzpatrick et al. [64] also recently reported a clinical and cognitive overlap between lvPPA and PCA in a single case study. Finally, Putcha et al. [65] recently showed that the word-retrieval profile of individuals with PCA is comparable to that of patients with the amnestic variant of AD (i.e., intact letter fluency but impaired category fluency and picture naming), while lvPPA patients demonstrated impairment across all tests. With respect to other cognitive functions, patients with lvPPA and PCA had similar levels of impairment in verbal episodic memory and verbal fluency, while their profile dissociated for visuospatial memory, visuospatial processing, executive functions and praxic domain [63].

From an anatomical point of view, the partial neuropsychological and neurolinguistic overlap between the two AD syndromes is not surprising. In brain imaging of PCA, atrophy or dysfunction is typically found bilaterally in the primary visual cortex, the visual association cortex, and the parietal lobes, while the anterior temporal and prefrontal cortical areas are largely spared [66]. In brain imaging of lvPPA, atrophy or dysfunction is predominantly found in the left temporo-parietal junction (posterior middle/superior temporal lobe and inferior parietal lobe) as well as in the left posterior cingulate, the precuneus, and the medial temporal lobe [67]. In a recent study of 56 individuals with lvPPA, Owens et al. [68] found significant grey-matter loss (left > right) in temporo-parietal regions with extension to the occipital lobes and the frontal regions. With the progression of the disease, the patterns of brain atrophy become less specific in PCA and lvPPA and converge across wide regions of the cortex [69].

Studies comparing neuroanatomical correlates of PCA and lvPPA have identified a large region of overlapping atrophy in the temporo-parietal network [18,70]. In addition, the temporo-parietal junction has been shown to be a common area of abnormalities in the two syndromes [28,71]. The temporo-parietal junction is a convergence area for multisensory integration and processing and receiving inputs from the thalamic, limbic, somatosensory, visual, and auditory cortices [72]. Furthermore, this area has bidirectional links with the prefrontal and temporal regions [73]. Lesions of the left angular gyrus (part of the temporo-parietal junction) are known to produce impairment of verbal short-term and verbal working memory [74] as well as various language deficits, including anomia [75], impaired sentence comprehension [76] and sentence repetition [77], alexia and agraphia [78]. As in previous studies on PCA and lvPPA, these linguistic abilities were affected in all three patients in the present study.

## 4. Conclusions

The specific characteristics of language impairment in PCA remain poorly described. In the present study we showed that PCA is characterized not only by visuospatial and visuoperceptual deficits but also by language impairment affecting sentence comprehension, word production, reading, and writing. According to us, this study is the first to specifically explore the functional origin of language impairment in PCA. The extensive description of the three cases has allowed us to show that most of these impairments are secondary to deficits of visuospatial and visuoperceptual processing (alexia, agraphia), verbal short-term and verbal working memory (impaired sentence comprehension and repetition), and executive functions (impaired verbal fluency). However, they also displayed primary language impairments affecting their ability to find words in conversation, use fluid and informative narrative speech, use the non-lexical route for reading nonwords, and spell words correctly.

Although the boundaries between PCA and lvPPA are less marked for language impairment, the two phenotypes of AD are markedly different with respect to deficits in visuospatial and visuoperceptual processing. Moreover, as pointed out by Crutch et al. [13], language impairment is not the most prominent clinical feature in PCA and is usually of a milder magnitude than in lvPPA. Nevertheless, the present study underlines that a systematic neurolinguistic assessment can be useful in the differential diagnosis of PCA. Such an assessment could also be the first step toward behavioral treatments of language disorders in PCA, such as those proposed for lvPPA [79,80,81]. In the present study, a clinical-like approach of assessment was used, so that different tests were used. In future studies, a comprehensive and uniformized assessment battery should be used to provide more convincing results. The results presented in this study are essentially descriptive. Going further in the characterization of primary and secondary language impairment associated with PCA would require the comparison with control participants and patients with AD.

## Figures and Tables

**Table 1:** Patients’ demographic data and medical and general cognitive test results.

Characteristics	Patient 1	Patient 2	Patient 3
Sex	M	F	M
Age (years)	63	74	64
Education (years)	8	16	9
Mother tongue	French	French	Portuguese
Languages spoken	French	French	Portuguese, English, French
Laterality	Right	Right	Left (forced to use the right hand)
Occupation	Artist	High school teacher (retired)	Forklift operator
PMHx and labs	No PMHx Normal labs	HTN Active alcohol intake (5/day) Normal labs	HTN Active alcohol intake (5/day) ↑ Cholesterol Otherwise, normal labs
Duration of symptoms (years)	8	6	2
Neurological examination	Normal	Normal	Slight decrease in eye blinking Empty gaze Slightly slow visual saccades Cogwheeling rigidity of the left arm
General cognitive screening	MoCA = 12/30	MoCA = 11/30 MMSE = 13/30	MoCA = 15/30 MMSE = 21/30
Neuroimaging	MRI (2014): Mild cortical atrophy and isolated microvascular lesions to the left corona radiata FDG-PET (2016): ↓ metabolism: Right posterior occipital region, bilateral posterior parietal cortex (right > left)	MRI (2017): T2/FLAIR white matter periventricular hyperintensities, volume loss in bilateral temporal lobes FDG-PET (2018): ↓ Metabolism of left polymodal associative areas especially parieto-occipital regions, asymmetry of primary visual areas (left > right)	CT scan (2018): Normal FDG-PET (2018): ↓ metabolism: Polymodal associative areas, posterior and prefrontal (left > right), left premotor cortex
Main complaints	↓ Visuospatial and visuoperceptual abilities Word-finding, reading, and writing difficulties	Word-finding and mental calculation difficulties	Difficulty expressing thinking Production of shorter sentences Difficulty parking forklifts in the warehouse

Note: CT = computerized tomography, FDG-PET = fluorodeoxyglucose-positron emission tomography, HTN = hypertension, MMSE = Mini-Mental State Examination, MoCA = Montreal Cognitive Assessment, MRI = magnetic resonance imaging, PMHx = past medical history. ↑ = increased; ↓ = decreased.

**Table 2:** Patients’ cognitive test results.

Cognitive Domain	Patient 1	Patient 2	Patient 3
Executive functions and short-term and working memory			
Executive functions	↓↓	↓↓	↓↓↓
Verbal short-term memory	Unimpaired	↓↓	↓↓
Verbal working memory	↓↓↓	↓↓↓	↓↓↓
Episodic memory			
Encoding process	Unimpaired	Unimpaired	Unimpaired
Consolidation process	Unimpaired	Unimpaired	Unimpaired
Retrieval process	↓	↓	↓
Visuospatial and visuoperceptual abilities			
Visuospatial abilities	↓↓↓	↓↓↓	↓↓↓
Visuoperceptual abilities	↓↓↓	↓	↓↓↓
Sensorimotor execution			
Ideomotor abilities	↓	↓	↓
Visuoconstructive abilities	↓↓↓	↓↓↓	↓↓↓
Oral-facial praxis	↓	↓	↓
Balint syndrome (ocular apraxia, simultanagnosia, visual ataxia)	Present	Present	Present
Gerstmann’s syndrome (agraphia, digital agnosia, acalculia, left and right confusion)	Present	Present	Present

Note: ↓ = mild impairment, ↓↓ = moderate impairment, ↓↓↓ severe impairment.

**Table 3:** Patients’ language test results.

Linguistic Domain	Patient 1	Patient 2	Patient 3
Semantic memory	Unimpaired	↓	Unimpaired
Oral comprehension			
Words	Unimpaired	↓	Unimpaired
Sentences	↓	↓	↓
Complex instructions	↓	↓	↓
Picture naming			
Performance	↓	↓↓↓	Unimpaired
Error type	Visual and visuo-semantic errors	Circumlocutions and visuo-semantic errors	Circumlocutions
Facilitation	Phonemic or syllabic cues	Phonemic or syllabic cues	
Verbal fluency			
Letter fluency	↓	↓↓↓	↓↓↓
Semantic fluency	↓	↓↓↓	↓↓↓
Repetition			
Words and nonwords	NA	Unimpaired	Unimpaired
Sentences	NA	↓ (for long sentences)	↓ (for long sentences)
Narrative discourse	Mildly tangential, word-finding difficulties	↓ Speech rate, word-finding difficulties, use of generic words, production of formal and phonological errors	Verbal aspontaneity, word-finding difficulties, short sentences, production of semantic and phonological errors
Reading	Unimpaired for words ↓ Nonwords ↓ Sentences (length effect) ↓ Arabic numbers Production of visual errors ↓ Comprehension	Unimpaired for words and nonwords but slow ↓ Arabic numbers (length effect) ↓ Comprehension (length effect)	Impaired word and nonword recognition Slow and jerky reading Line breaks and word omissions Numerous visual errors ↓↓↓ Comprehension
Writing	Peripheral agraphia: Distorted letters Central agraphia: Production of non-phonologically plausible errors (letter omissions and substitutions)	Peripheral agraphia: Distorted letters Central agraphia: Production of non-phonologically plausible errors (letter omissions and substitutions)	Peripheral agraphia: Distorted letters, difficulty holding a pencil Central agraphia: Production of phonologically and non-phonologically plausible errors

Note: ↓ = mild impairment, ↓↓ = moderate impairment, ↓↓↓ severe impairment; NA = Not Administered.
